# Application of A Novel Three-dimensional Printing Genioplasty Template System and Its Clinical Validation: A Control Study

**DOI:** 10.1038/s41598-017-05417-7

**Published:** 2017-07-14

**Authors:** Biao Li, Hongpu Wei, Feini Zeng, Jianfu Li, James J. Xia, Xudong Wang

**Affiliations:** 1 Resident, Department of Oral and Craniomaxillofacial Surgery, Shanghai 9th People’s Hospital, Shanghai Jiaotong University College of Medicine, Shanghai, China; 2 Researcher, Shanghai Key Laboratory of Stomatology, Shanghai, China; 3 Engineer, Department of Oral and Craniomaxillofacial Surgery, Shanghai 9th People’s Hospital, Shanghai Jiaotong University College of Medicine, Shanghai, China; 4 Postdoctoral Fellow, Surgical Planning Laboratory, Department of Oral and Maxillofacial Surgery, Houston Methodist Research Institute, Houston, Texas, USA; 5 Guest Professor, Department of Oral and Craniomaxillofacial Surgery, Shanghai 9th People’s Hospital, Shanghai Jiaotong University College of Medicine, Shanghai, China; 6 Director of Surgical Planning Laboratory and Professor of Oral and Maxillofacial Surgery, Institute for Academic Medicine, Houston Methodist Hospital, Texas, USA; 7 Professor of Surgery (Oral and Maxillofacial Surgery), Weill Medical College, Cornell University, New York, USA; 8 Acting Chair and Professor, Department of Oral and Craniomaxillofacial Surgery, Shanghai 9th People’s Hospital, Shanghai Jiaotong University College of Medicine, Shanghai, China; 9 Professor, Shanghai Key Laboratory of Stomatology, Shanghai, China

## Abstract

The purpose of this control study was to assess the accuracy and clinical validation of a novel genioplasty template system. Eighty-eight patients were enrolled and divided into 2 groups: experimental group (using genioplasty templates) and control group (without genioplasty templates). For the experimental group, the templates were designed based on computerized surgical plan and manufactured using three-dimensional printing technique. The template system included a cutting guide and a pair of repositioning guides. For the control group, traditional intraoperative measurements were used without genioplasty templates. The outcome evaluation was completed by comparing planned outcomes with postoperative outcomes. Linear and angular differences for the chin was measured and reported using root mean square deviation (RMSD) and the Bland-Altman method. All surgeries were successfully completed. There was no difficulty to use genioplasty templates. For the experimental group, the largest RMSDs were 1.1 mm in anteroposterior direction and 2.6° in pitch orientation. For the control group without templates, the largest RMSDs were 2.63 mm in superoinferior direction and 7.21° in pitch orientation. Our findings suggest that this genioplasty template system provides greater accuracy in repositioning the chin than traditional intraoperative measurements, and the computerized plan can be transferred accurately to the patient for genioplasty.

## Introduction

Chin plays an important role in the lower facial harmony and balance^1^. Chin deformity is commonly associated with dentofacial deformities. Genioplasty is a surgical procedure widely used to correct chin deformities^2^. It is critical to make an optimal surgical plan for the genioplasty because the location of the osteotomy and the movement of the bony segment directly impact surgical outcome^3^. Traditionally, genioplasty is performed based on surgeon’s intraoperative assessment. When the genioplasty is only required for the patient, the repositioning of the chin could be traditionally determined based on clinical experience. However, when the genioplasty combined with bimaxillary orthognathic surgery is required for the patient, it would be difficult for the surgeon to imagine the chin position following bimaxillary surgery and to determine the repositioning plan for genioplasty. Without a detailed surgical plan and a reliable transferring modality, this task can be difficult as it is heavily depending on the surgeon’s experience. Besides, soft tissue edema after bimaxillary surgery will make it more difficult to determine the surgical plan based on the intraoperative evaluation of the chin, which will lead to high demand for clinical experience to achieve the optimal postoperative outcome. The surgical outcome is often less than ideal^4, 5^.

With the advances in computer-aided surgical simulation (CASS) technology, surgeons are now able to simulate the entire orthognathic surgery and test various surgical plans in a computer until the best possible outcome is achieved^6–8^. Computer-aided designing/computer-aided manufacturing (CAD/CAM) surgical templates have been used to transfer the computerized surgical plan to the patient at the time of the surgery. These templates can guide the surgeon to accurately perform the osteotomy and move the bony segment to the desired position exactly as planned^4, 7, 9^.

It has been largely documented that the carefully planned maxillary and mandibular positions can be accurately transferred to the patient at the time of the surgery using CAD/CAM guides^4, 5^. A preliminary study published by our group showed that the surgical template technique could provide a reliable method of planning transfer for segmental genioplasty^10^. To date, there were only a few studies with small sample sizes on the use of genioplasty templates^4, 10^. However, the technique and the accuracy of transferring planned genioplasty at the time of the surgery still remained uncertain.

In this paper, the authors completed a control study to develop and validate their novel genioplasty templates system for monoblock genioplasty.

## Materials and Methods

Eighty-eight patients (median age: 25 years; range: 18–30 years) with dentofacial deformities were enrolled in this study from January 2014 to April 2016 at our department. Patient’s inclusion criteria were: 1) patients who were scheduled to undergo monoblock genioplasty immediately following bimaxillary orthognathic surgery; 2) patients who were scheduled to undergo a CT scan as a part of their diagnosis and treatment; and 3) patients who agreed to participate in this study. Exclusion criteria were: 1) patients with craniofacial syndrome; 2) patients with previous osseous genioplasty; 3) patients with previous mandibular trauma; and 4) patients requiring segmental genioplasty.

This study included two groups: the experimental group (using the genioplasty template system) and the control group (using conventional method without genioplasty templates). The study was approved by Shanghai 9^th^ People’s Hospital IRB. Informed consent was obtained prior to the study. All methods were performed in accordance with the relevant guidelines and regulations of our hospital.

### Surgical Planning Following CASS Protocol

A preoperative computed tomography (CT) scan of patient’s head was acquired with 1.25mm of slice thickness in supine position (GE Healthcare, Fairfield, USA). A wax occlusal bite was used to slightly separate the maxilla and mandible and maintain the centric relation. The CT data were imported into planning software (ProPlan 2.0, Materialise NV, Leuven, Belgium) to generate three-dimensional (3D) maxillary and mandibular models. The digital dental models were generated by scanning a set of stone dental models using a high-resolution laser surface scanner (SmartOptics AS, Bochum, Germany). The digital dental models were then integrated into the 3D skull model to replace the less-than-accurate CT teeth. This resulted in a computerized composite skull model with accurate rendition of both the bony structures and the teeth^11^.

A patient-specific reference frame was established based on the composite skull model^4^. In this study, *nasion* was defined as the origin of the reference frame for the composite skull model, with *X* axis running in mediolateral direction, *Y* axis in anteroposteriorly direction and *Z* axis in inferosuperior direction. The midsagittal (YOZ) plane was a vertical plane that best divides the face into right and left halves based on clinical examination and neutral head posture (NHP) records^12, 13^. The axial (XOY) plane was the horizontal plane passing through *nasion*, dividing the head to the upper and lower parts. The coronal (XOZ) plane was the vertical plane that was perpendicular to the other two planes.

After the reference frame of the composite skull model was established, bimaxillary orthognathic surgery was simulated in the computer by surgeons following the standard CASS planning routine^4, 8, 11–13^. During the genioplasty simulation, the inferior nerve cannels were marked on the mandibular model before the osteotomy planning to avoid inferior alveolar nerves injury. Then the repositioning of the chin segment was planned based on clinical examination, cephalometric analysis and 3D measurements to achieve the ideal outcome.

### Design and Intraoperative Utilization of Genioplasty Templates System

Once the surgical plan was finalized, the 3D models of the bony segments, in both their initial and the planned positions, were imported into CAD/CAM software (Geomagic Studio, Geomagic, NC, US) to design the surgical splints and genioplasty templates.

The CAD/CAM surgical splints were designed to position the maxillary Le Fort I and mandibular distal segments following a routine procedure for both groups^12^. The surgeons used intraoperative measurements to reposition the chin segment in control group. For experimental group, the genioplasty template system was designed to transfer the genioplasty plan to operative filed. All the designs, i.e., the geometry and placement position of all the surgical templates, were approved by the surgeons prior to the surgery. Stereolithography (STL) files of all splints and templates were exported for manufacturing process using a 3D printing machine (3D Systems, Rock Hill, USA) with photosensitive resin.

### Design of Genioplasty Template System

Our genioplasty templates system includes 2 parts: a cutting guide and a pair of repositioning guides. The cutting guide is designed to assist the surgeon to perform the osteotomy and predrill the screw holes which in conjunction with the use of the following reposition guides assisted the chin segment repositioning as planned. All the 3D models of the bony segments were located at their original positions during the designing process of the cutting guide.

**Figure 1:**
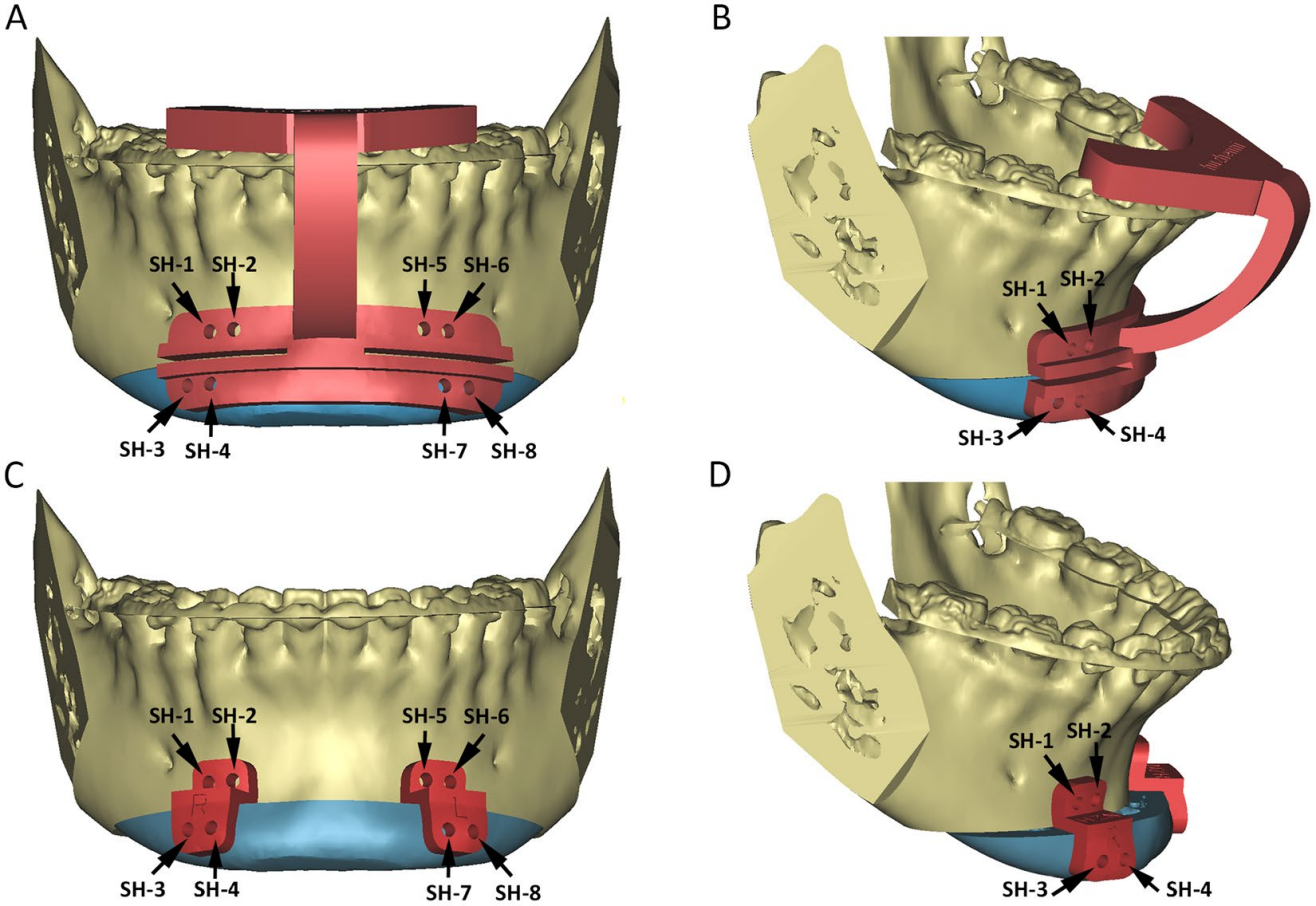
(**A**,**B**) The upper portion of the cutting guide is designed like a dental splint. The lower portion of the guide is designed to indicate the cutting lines and the trajectory of the cutting plane. Eight screw-hole drilling guides (black arrows) are designed on both sides of the osteotomy cutting lines to provide stable bony reference landmarks. (**C**) After the osteotomy is completed, a pair of the repositioning guides are installed. The upper portion of each guide is attached to the distal mandible using the original two screw holes (SH-1 and SH-2 on the right side; SH-5 and SH-6 on the left side). The lower portion of each guide includes two repositioning screw holes in their planned final positions (SH-3 and SH-4 on the right side; SH-7 and SH-8 on the left side). (**D**) The new location of the screw holes on the repositioning guide will automatically bring the chin segment into its planned final position as the screws are placed into the appropriate screw holes. (Chin segments are marked in teal; SH: screw hole).

The upper portion of the cutting guide was designed like a tooth-borne splint, serving as a locking mechanism to accurately install the cutting guide at the unique position on the mandibular teeth^4, 11^. The lower portion of the guide was designed to mark the position and angle of the osteotomy plane (Fig. 1A,B). The cutting guide was not extended to the mental foramen area to avoid nerve injure (Fig. 1A). In addition, the cutting guide was also used as a drilling guide for the fixation screws. Eight screw holes were designed on both sides of the osteotomy lines, 2 pairs on the left side and 2 pairs on the right (Fig. 1A). These 8 screw holes not only were used for temporary fixing the cutting guide, but also served as the repositioning bony reference landmarks for the next step. Finally, the upper and lower portions of the cutting guide was connected by a solid bar, which was designed like a curved bridge to avoid interference between the guide and orthodontic braces (Fig. 1B).

The repositioning guide set included a pair of guides, one on the left and the other on the right. They were used to reposition the chin segment to its final position (Fig. 1C). This design was to avoid any possible interference between the guides and titanium genioplasty plates. During the designing process, the mandibular and chin segments were located at their finally planned positions. The upper portion of the repositioning guide was designed to fit the bony surface of the distal mandible above the osteotomy line, while the lower portion of the repositioning guide was designed to fit the bone surface of the chin segment at final position. In addition, the screw holes on the reposition guides were designed to be consistent with the holes on corresponding models that were determined by the cutting guide before. The locations of the screw holes on the repositioning guides automatically brought the chin segment to its planned position as the correspondent holes were matched and the screws were placed into the appropriate screw holes (Fig. 1D).

### Surgery

**Figure 2:**
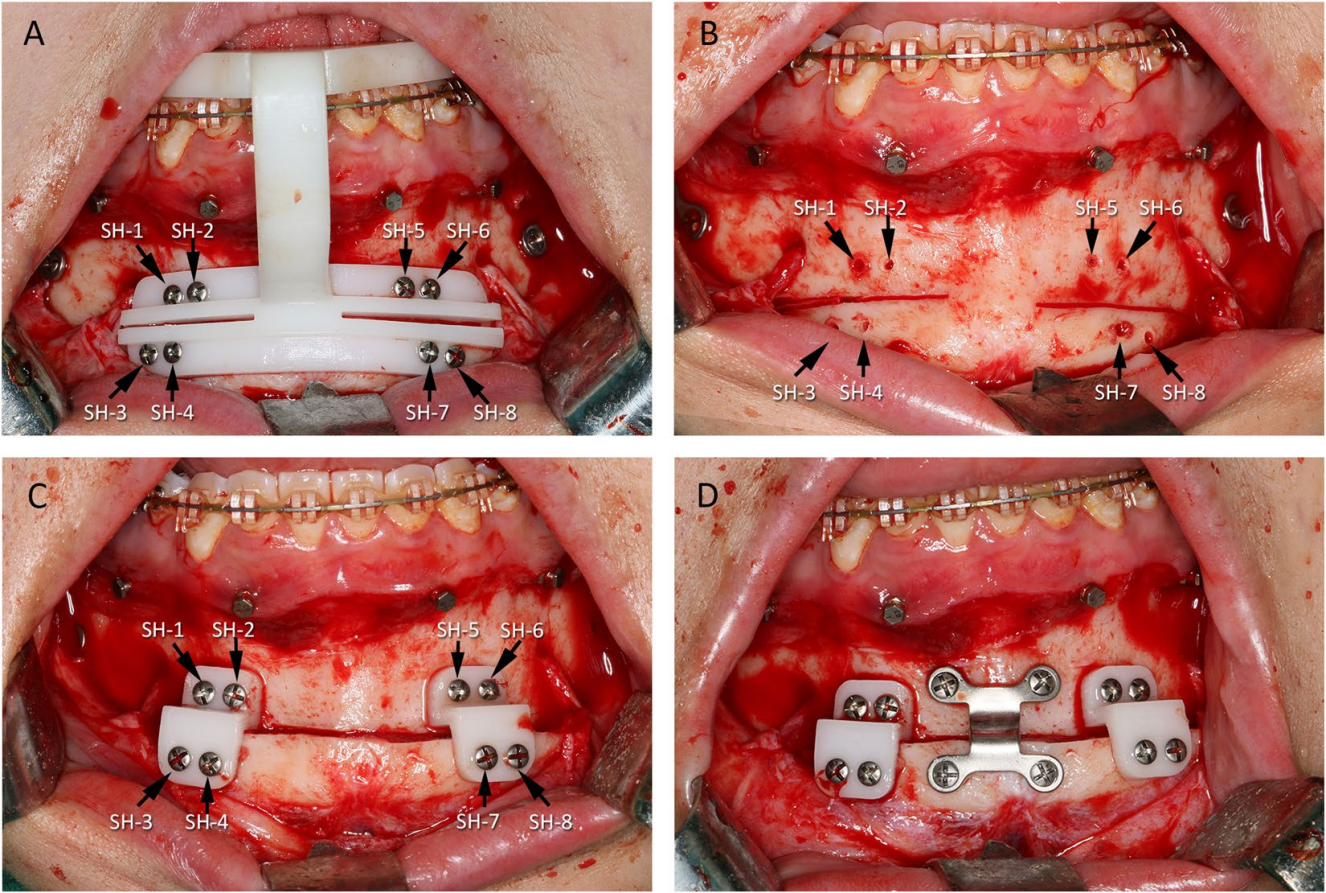
(**A**) During the operation, the cutting guide was positioned on the mandibular dentition exactly as planned. The lower portion of the guide was attached firmly to the chin by eight screws using the drilling holes. The resection margins guided the saw blade to perform the osteotomy as planned. (**B**) The cutting guide and the screws were removed to complete the osteotomy after the cutting lines had been marked and the screw holes had been drilled. (**C**) A pair of the repositioning guides were installed on the distal mandible by alignment with the previously drilled screw holes on the distal mandible. When the four corresponding screw holes on both sides of the chin segment and the guide were aligned, the chin segment was automatically moved to its final planned position and secured temporarily. (**D**) The chin segment was stabilized by rigid fixation. Then, the guide and associated screws were removed. (SH: screw hole).

During the surgery, the CAD/CAM surgical splints were used to routinely place the maxilla and distal mandible to the final planned positions for both group patients^11^. For the genioplasty, surgical template system was used to reposition the chin segment in experimental group. During the operation, the anterior surface of the chin was exposed as in the routine procedure via intraoral approach. The cutting guide was first attached to the mandibular dentition as planned. The 8 screw holes were then drilled based on drilling holes on the cutting guide, and the cutting guide was temporarily fixed onto the mandible (Fig. 2A). Once the osteotomy/ostectomy lines were marked by a surgical saw, the cutting guide were removed and osteotomy completed routinely (Fig. 2B).

After the osteotomy was completed, the reposition guides were installed to reposition the chin segment as planned. Firstly, the upper portion of the repositioning guides was firmly attached on the distal mandible above the osteotomy line using the corresponding screw holes on the guide and the bone. Next, the position of the chin segment was aligned till all the corresponding screw holes on the chin and the lower portion of the reposition guides were matched (Fig. 2C). Afterwards, as the screws were placed and tightened, the chin segment was automatically moved to its final planned position and secured. Finally, after the chin segment was stabilized by rigid fixation (Fig. 2D), the guides and associated screws were removed.

In the control group, the genioplasty was accomplished without using genioplasty templates^1^. Surgeons used intraoperative measurements to reposition the chin segment based on preoperative planning.

### Outcome Evaluation

A postoperative CT scan was acquired within 6 weeks after the surgery. It represented the actual surgical outcomes. The accuracy of the chin segment was assessed by comparing the actual postoperative outcomes to the planned ones. Both planned and postoperative models were imported into CAD software (3-Matic, Materialise NV, Leuven, Belgium).

**Figure 3:**
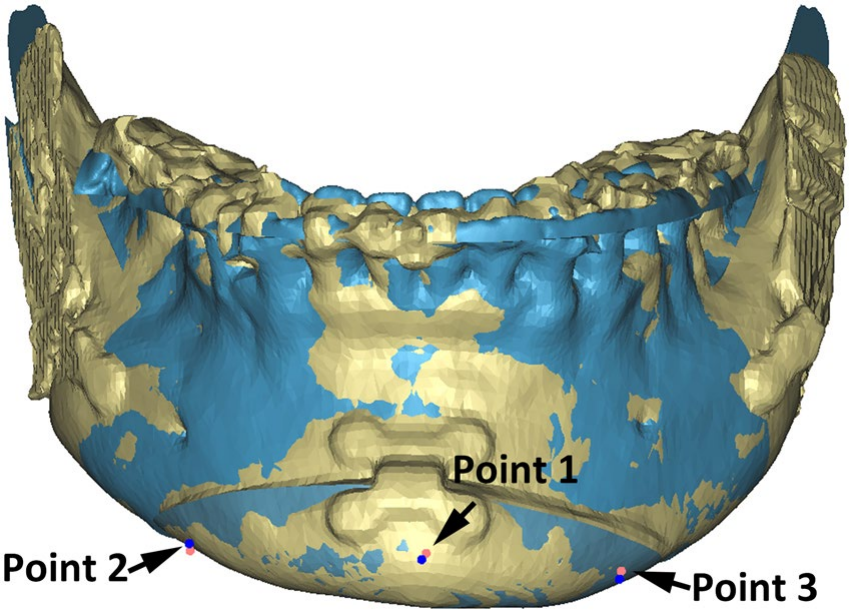
Three corner points of the chin segment were digitized on both planned and postoperative models to define the position and orientation of the chin segment. The landmarks utilized were: the pogonion (Point 1) and bilateral corner point on the buccal surface of the chin segment (Point 2 and Point 3). The landmarks on the planned models were marked in red, and the landmarks on the postoperative models were marked in blue. The planned models were showed in blue, and the postoperative models were showed in yellow.

We used the coordinates of three independent points to define the position and orientation of the chin in 3D space^4^. Three landmark points of the chin segment, the pogonion and bilateral corner point on the buccal surface, were digitized on both planned and postoperative models (Fig. 3). A ‘reversed’ routine developed by Xia *et al*.^6^ and Hsu *et al*.^4^ was used to ensure the correspondence of landmarks between the planned and the postoperative chin segments. The coordinates of all landmarks and the centroid of the chin segment object was used to measure the differences between the plan and postoperative results.

The postoperative mandibular models were then registered to the planned distal mandible. Using our previously validated method^4, 6^, the registration was completed by superimposing the area of each model that was not moved by surgery, i.e. the distal mandibular region above the osteotomy of the genioplasty. The planned models were kept static, and served as targets. During the registration, all the landmarks and the chin segments were hidden. Only the region of the distal mandible above the cutting line was visualized. These precautions were done to avoid operator’s bias. The postoperative CT models were registered to the planned models using the surface-best-fit method. Finally, after the registration was completed, all hidden landmarks for the chin segment were displayed and their raw coordinates were recorded.

The linear differences between the planned and postoperative outcomes were calculated in x, y and z dimensions. The angular differences were also calculated in pitch (the rotation around the x axis), roll (the rotation around the y axis), and yaw (the rotation around the z axis)^4, 14^. The calculation of the angular differences was automatically completed using a custom MATLAB program (MathWorks, Natick, MA, USA).

### Statistical Analysis and Data Presentation

The accuracy of the chin segment position was reported using two methods. The first method was the root mean square deviation (RMSD), which was used to report linear and angular differences for the accuracy of our genioplasty templates. The second reporting approach was the Bland-Altman method to assess measurement agreement^15^. The independent *t* test was used to assess the significance of differences in all directions between the experimental and control groups. Probabilities of less than 0.05 were accepted as significance, and the analysis was made with the help of the SPSS 13 (SPSS Inc., Chicago, IL, USA).

To help interpret the results of the accuracy measurements for the chin segment, we considered positional differences of less than 1 mm to be clinically insignificant^16, 17^. We also considered an orientational differences of less than 4° to be clinically inconsequential^18^.

## Results

All genioplasty were successfully completed. All the patients healed uneventfully with no wound dehiscence. All temporary postoperative nerve paresthesia, if any, was fully recovered within 1–3 months. There was no difficulty to install and use both cutting and repositioning guides. Due to the curved design of the cutting guide, and slim and standalone design of the reposition guides, the interference between the guides and orthodontic braces or titanium genioplasty plates was never encountered. In addition, there were no signs of abnormal bleeding, mental nerve damage, and template breakage for experimental group.

The linear and angular RMSD differences between the planned and the postoperative chin segments are presented in Table 1. For the experimental group with the use of genioplasty template, the largest linear discrepancy between the planned and the postoperative chin segments occurred in anteroposterior direction: RMSD was 1.1 mm, and Bland-Altman lower and upper limits were −2.3 mm and −0.6 mm (Tables 1 and 2). It indicated that the postoperative chin segments were unexpectedly located posteriorly to the planned ones. The largest angular discrepancy occurred in pitch orientation: RMSD was 2.6°, and Bland-Altman lower and upper limits were −5.77° and 2.75° (Tables 1 and 3). It indicated that the chin segments had an unplanned rotation around the mediolateral (x−) axis. For the control group without genioplasty template, the largest linear discrepancy of the chin position occurred in vertical direction: RMSD was 2.63 mm, and Bland-Altman lower and upper limits were −4.48 mm and 5.68 mm (Tables 1 and 2). The largest angular discrepancy occurred in pitch orientation: RMSD was 7.21°, and Bland-Altman lower and upper limits were −15.89° and 7.89° (Tables 1 and 3).

**Table 1:** Rood mean square deviation (RMSD) of Positional and Orientational Differences between the Planned and Postoperative Outcomes.

Chin	Positional Difference (mm)		Orientation Difference (degree)	
Without template	Mediolateral	1.45	Pitch	7.21
	Anteroposterior	2.13	Roll	2.72
	Superoinferior	2.63	Yaw	2.92
With template	Mediolateral	0.62	Pitch	2.63
	Anteroposterior	1.11	Roll	1.77
	Superoinferior	0.84	Yaw	1.51

**Table 2:** Bland-Altman’s Accuracy Estimate of Positional Differences between the Planned and Postoperative Outcomes (mm).

Chin		Mean	SD	Positional Difference (95% CI) (mm)			
				Lower Limit		Upper Limit	
Without template	Mediolateral	0.04	1.47	−2.84	(−4.57 to −2.07)	2.91	(2.14 to 3.68)
	Anteroposterior	−1.22	1.76	−4.68	(−6.41 to −3.75)	2.24	(1.31 to 3.16)
	Superoinferior	0.60	2.59	−4.48	(−6.21 to −3.12)	5.68	(4.31 to 7.04)
With template	Mediolateral	−0.01	0.62	−1.23	(−2.96 to −0.90)	1.21	(0.88 to 1.54)
	Anteroposterior	−0.84	0.74	−2.28	(−4.02 to −1.90)	0.60	(0.21 to 0.99)
	Superoinferior	0.02	0.85	−1.64	(−3.37 to −1.20)	1.67	(1.23 to 2.12)

**Table 3:** Bland-Altman’s Accuracy Estimate of Orientational Differences between the Planned and Postoperative Outcomes (degrees).

Chin		Mean	SD	Orientation Difference (95% CI) (degree)			
				Lower Limit		Upper Limit	
Without template	Pitch	−4.00	6.06	−15.89	(−17.62 to −12.69)	7.89	(4.69 to 11.08)
	Roll	−0.60	2.69	−5.87	(−7.6 to −4.45)	4.67	(3.26 to 6.09)
	Yaw	−0.70	2.87	−6.32	(−8.05 to −4.81)	4.93	(3.42 to 6.44)
With template	Pitch	−1.51	2.17	−5.77	(−7.50 to −4.63)	2.75	(1.61 to 3.90)
	Roll	0.33	1.76	−3.11	(−4.84 to −2.18)	3.77	(2.85 to 4.70)
	Yaw	−0.04	1.53	−3.04	(−4.77 to −2.23)	2.95	(2.14 to 3.75)

The worse outcomes were found in the control group not using the genioplasty templates. In all 6 measurements of the chin position and orientation, the result of independent *t* test showed that there was statistically significant difference between the groups with and without the genioplasty templates.

## Discussion

An optimal surgical plan and accurate intraoperative performing is the premise of achieving a satisfied postoperative outcome for genioplasty. In this study, we developed this genioplasty template system that is designed to guide both chin osteotomy and repostioning. The result of the quantative analysis showed the accuracy of the genioplasty template system is clincially acceptable. The genioplasty template system provides greater accuracy in repositioning the chin segment than the traditional method without using the genioplasty templates.

There are some reports on the use of CAD/CAM genioplasty templates for monoblock genioplasty^4, 7, 11^. However, most of these templates were cumbersomely designed, which could increase the difficulty of intraoperative applying. Our genioplasty templates system consists of two sets of surgical guides: a cutting guide and a pair of reposition guides. It has the following advantages. First, the cutting guide could assist surgeons precisely duplicating the osteotomy and ostectomy as planned. As inferior alveolar nerve canal is marked out in preoperative plan, the osteotomy is guided to avoid the inferior alveolar nerve injure. Second, the screw holes, which indicated by the cutting guide, served as reference landmarks for repositioning the bony segments in conjunction with the use of the repositioning guides. The repositioning guides are designed to automatically reposition the chin segment to the final planned position using these screw holes. Third, in order to be adapted easily in the limited surgical field, the repositioning guides are designed to be slim and separated right and left guides. Fourth, it is much easier for surgeons to bend and adapt titanium genioplasty plates intraoperatively as the chin segment is stabilized by the repositioning guides. Finally, because the chin position is planned preoperatively in the computer and the plan can be accurately transferred to the patient at the time of the surgery, the repeatedly intraoperative measurement and visual assessment are no longer needed. It makes possible for a young surgeon with less clinical experience to accurately perform genioplasty and achieve more ideal surgical outcomes.

Positional difference in anteroposterior direction (1.1 mm) is slightly larger than expected in the group with the use of the genioplasty template. The largest angular difference (2.6°), although within our expectation, is also occurred in pitch. One hypothesis to explain this phenomenon is that the repositioning guides are only applied to the buccal side of the chin segment. The retraction force of the soft tissue (mainly from the suprahyoid depressor muscles) is considerably large, causing the slim-designed repositioning guides slightly deformed. Although visually unnoticeable, the deformation of the repositioning guides may directly result in an unexpected posterior displacement and pitch rotation of the chin segment before the rigid fixation is applied. A second hypothesis is that the titanium genioplasty plate is not rigid enough to resist the retraction force from the suprahyoid depressor muscles after patient wakes up from general anesthesia. Nonetheless, the accuracy is still considered clinically acceptable.

In the control group, the differences between the planned and actual outcomes were larger than the accepted clinical thresholds. The largest differences in control group existed in superoinferior directions and in the pitch and yaw orientations. One possible reason is that it is difficult to accurately control vertical translation and pitch rotation of the chin segment freehand. Although marker lines could provide valuable assistance in mediolateral translation of the chin, they could barely help the surgeon to control the rotation of chin segment.

This study indicated that the genioplasty templates could increase the accuracy of genioplasty. However, the use of the genioplasty template was associated with a small increase in surgical times and a modest increase in cost, which would be further studied in future.
